# Reply to Nishimura, “Questions regarding mortality and infection source in deep-seated infections”

**DOI:** 10.1128/aac.01716-25

**Published:** 2026-03-02

**Authors:** Nicole Slain, Ashlan J. Kunz Coyne

**Affiliations:** 1 University of Kentucky HealthCare 177468, Lexington, Kentucky, USA; 2 University of Kentucky College of Pharmacy 15511, Lexington, Kentucky, USA; University of Fribourg, Fribourg, Switzerland

## REPLY

Though previous studies have suggested cefepime as a suitable option for infections caused by AmpC-producing *Enterobacterales* (AmpC-E), this study focuses on patients with deep-seated infections, where challenges, such as the inoculum effect, and pharmacokinetic considerations are more pronounced.(1) The question regarding 30-day mortality rates in comparison to 60- or 90-day mortality rates in the cefepime group is a noted observation.(2) As mentioned in the discussion, the 30-, 60-, and 90-day mortality rates were similar between patients in the cefepime and carbapenem groups. Although the 30-day mortality rate was numerically higher in the cefepime group, this difference was not statistically significant. It is also important to clarify that these mortality endpoints represent separate, non-cumulative time windows, so deaths occurring within 30 days are not included again in the 60- or 90-day analyses. Additionally, the 30-day clinical failure endpoint incorporates both mortality and infection recurrence, whereas recurrence was not evaluated at 60 or 90 days. This distinction in endpoint definitions explains why differences were observed in 30-day clinical failure, while later mortality measures remained similar.

Deep abscesses in this study were defined, per the Materials and Methods section, as infections occurring in normally sterile compartments, such as intra-abdominal, thoracic, or pelvic spaces. There was no statistically significant difference in the frequency of deep abscesses between patients treated with cefepime and those treated with carbapenems. Because there was no significant difference between groups, and the study was not powered to evaluate outcomes by individual anatomic sites, we did not further subclassify these abscesses beyond the predefined categories. In many cases of deep abscesses, empiric anaerobic coverage is warranted because anaerobes may be difficult to isolate and may not grow in culture, and this has been common practice at our institution. Most patients were evaluated by an infectious diseases physician, and adjunct anaerobic therapy is frequently utilized in these scenarios. However, data regarding the use of empiric anaerobic coverage in addition to cefepime were not collected, and the absence of these data, particularly within the cefepime group, represents a limitation of this study.

The reviewer is correct that not all deep-seated infections require a procedural source control intervention. As defined in the Materials and Methods section, source control in this study included surgical or percutaneous drainage, debridement, or valve replacement or removal when these procedures were performed to eliminate the infectious focus. Deep-seated infections in our cohort, such as vertebral osteomyelitis or certain bone and joint infections, may be managed medically without a procedural intervention depending on clinical presentation, and these patients were included if they received antimicrobial therapy consistent with evidence-based treatment practices for that infection type. Source control was not required for study inclusion, and the study did not classify infections by whether source control was clinically indicated. For this reason, we cannot report the number of cases in which source control was needed versus the number in which it was performed. The study captured only whether a source control procedure occurred and whether it met predefined criteria for success based on clinical, microbiologic, radiographic, or clinician-assessed improvement. The inability to distinguish between cases in which source control was indicated and those in which it was not represents a limitation of the retrospective study design.
